# Clinical assistant decision-making model of tuberculosis based on electronic health records

**DOI:** 10.1186/s13040-023-00328-y

**Published:** 2023-03-16

**Authors:** Mengying Wang, Cuixia Lee, Zhenhao Wei, Hong Ji, Yingyun Yang, Cheng Yang

**Affiliations:** 1 State Key Laboratory of Media Convergence and Communication, Communication University of China, No .1 Dingfuzhuang East Street, Chaoyang District, Beijing, China; 2 Peking University Third Hospital, Beijing, China; 3 Goodwill Hessian Health Technology Co.Ltd, Beijing, China

**Keywords:** Electronic health records, Clinical decision support, Predictive model, Tuberculosis

## Abstract

**Background:**

Tuberculosis is a dangerous infectious disease with the largest number of reported cases in China every year. Preventing missed diagnosis has an important impact on the prevention, treatment, and recovery of tuberculosis. The earliest pulmonary tuberculosis prediction models mainly used traditional image data combined with neural network models. However, a single data source tends to miss important information, such as primary symptoms and laboratory test results, that is available in multi-source data like medical records and tests. In this study, we propose a multi-stream integrated pulmonary tuberculosis diagnosis model based on structured and unstructured multi-source data from electronic health records. With the limited number of lung specialists and the high prevalence of tuberculosis, the application of this auxiliary diagnosis model can make substantial contributions to clinical settings.

**Methods:**

The subjects were patients at the respiratory department and infectious cases department of a large comprehensive hospital in China between 2015 to 2020. A total of 95,294 medical records were selected through a quality control process. Each record contains structured and unstructured data. First, numerical expressions of features for structured data were created. Then, feature engineering was performed through decision tree model, random forest, and GBDT. Features were included in the feature exclusion set as per their weights in descending order. When the importance of the set was higher than 0.7, this process was concluded. Finally, the contained features were used for model training. In addition, the unstructured free-text data was segmented at the character level and input into the model after indexing. Tuberculosis prediction was conducted through a multi-stream integration tuberculosis diagnosis model (MSI-PTDM), and the evaluation indices of accuracy, AUC, sensitivity, and specificity were compared against the prediction results of XGBoost, Text-CNN, Random Forest, SVM, and so on.

**Results:**

Through a variety of characteristic engineering methods, 20 characteristic factors, such as main complaint hemoptysis, cough, and test erythrocyte sedimentation rate, were selected, and the influencing factors were analyzed using the Chinese diagnostic standard of pulmonary tuberculosis. The area under the curve values for MSI-PTDM, XGBoost, Text-CNN, RF, and SVM were 0.9858, 0.9571, 0.9486, 0.9428, and 0.9429, respectively. The sensitivity, specificity, and accuracy of MSI-PTDM were 93.18%, 96.96%, and 96.96%, respectively. The MSI-PTDM prediction model was installed at a doctor workstation and operated in a real clinic environment for 4 months. A total of 692,949 patients were monitored, including 484 patients with confirmed pulmonary tuberculosis. The model predicted 440 cases of pulmonary tuberculosis. The positive sample recognition rate was 90.91%, the false-positive rate was 9.09%, the negative sample recognition rate was 96.17%, and the false-negative rate was 3.83%.

**Conclusions:**

MSI-PTDM can process sparse data, dense data, and unstructured text data concurrently. The model adds a feature domain vector embedding the medical sparse features, and the single-valued sparse vectors are represented by multi-dimensional dense hidden vectors, which not only enhances the feature expression but also alleviates the side effects of sparsity on the model training. However, there may be information loss when features are extracted from text, and adding the processing of original unstructured text makes up for the error within the above process to a certain extent, so that the model can learn data more comprehensively and effectively. In addition, MSI-PTDM also allows interaction between features, considers the combination effect between patient features, adds more complex nonlinear calculation considerations, and improves the learning ability of the model. It has been verified using a test set and via deployment within an actual outpatient environment.

## Introduction

Tuberculosis is a common and extremely dangerous infectious disease. Unless treated in time, it may lead to pulmonary failure and even be life-threatening. If a patient with infectious pulmonary tuberculosis is not treated, he or she can infect an average of 10–15 healthy people in one year [1]. According to the global tuberculosis report (2019) released by WHO the positive rate of the global average TB etiology (including bacteriology and analytical biology) is 55% [2]. From 2015 to 2019, the positivity rate of etiology in China increased from 31 to 47%, but it is still lower than the global average [2]. Although CT and other imaging methods can quickly diagnose tuberculosis, the imaging manifestations of some cases are atypical. Isolation of *Mycobacterium tuberculosis* from sputum culture is the main method for the diagnosis of tuberculosis, but the culture cycle of *Mycobacterium tuberculosis* is long and the positivity rate is low. Sputum cultures of some patients show up as negative (bacteria-negative tuberculosis) [3]. Tuberculin tests are widely used in the diagnosis of *Mycobacterium tuberculosis* infection, but they cannot distinguish between the natural infection of *Mycobacterium tuberculosis* and the immune response after BCG vaccination. In China, 34.61% of hospitals are primary medical institutions [4, 5]. The main examinations for pulmonary tuberculosis include X-ray examination, direct smear examination of acid-fast bacilli, tuberculin skin test (TST), and pleural effusion examination. Among the medical institutions, 8.46% were large general hospitals, and the main examinations included chest CT, tuberculosis culture, interferon gamma release assay (IGRA) and tuberculosis antibody detection, PCR technology, bronchoscopy, or other histopathological examinations [6]. Note that IGRA, PCR, and other methods are only used in large general hospitals in Chinese medical institutions, and cannot be widely implemented throughout the country [7, 8].

Therefore, there is an urgent need for rapid auxiliary diagnostic tools for tuberculosis, especially bacterial negative tuberculosis. The diagnosis of tuberculosis with positive etiology is relatively clear, while the diagnosis of tuberculosis with negative etiology lacks clear laboratory test support. A doctor needs to make a diagnosis based on the patient’s overall clinical manifestations, including the patient’s contact history of tuberculosis, clinical symptoms, and imaging features. However, owing to the lack of clinical experience of some doctors and atypical clinical manifestations, missed diagnosis and misdiagnosis are inevitable. Early and timely auxiliary diagnostic tools can effectively control the spread of pulmonary tuberculosis and improve the effectiveness of diagnosis and treatment [9]. Neural networks have been widely used in clinical decision-making, such as brain tumor detection [10], citric acid poisoning monitoring, early warning in ICUs [11], and bone age assessment [12]. Studies [13, 14] have also verified the effectiveness of neural networks in the real-world clinical auxiliary effect of the hospital based on the time of diagnosis and the length of hospital stay. Therefore, it is feasible to apply neural networks for clinical auxiliary prediction.

In earlier times, tuberculosis early warnings were based on questionnaire evaluations [15]. However, the information thus gathered could not be automatically combined with the actual medical records of patients, and there was a disconnect from real clinical setups. In addition to writing medical records, doctors needed to fill in the evaluation questionnaire independently, which increased their workload. A quicker low-cost method was needed to quickly identify tuberculosis within a clinical practice setup. Lee [16] proposed an image-based tuberculosis prediction model. The automatic detection algorithm (DLAD) was used to predict tuberculosis within 19 samples. The positive predictive values (PPVs) and negative predictive values (NPVs) were 0.959 and 0.997. Another study [17, 18] demonstrated that DLAD has a good classification performance in active tuberculosis under a high incidence rate (i.e., TB incidence rate of 9.1%), with the area under the receiver operating characteristic curve (AUC) being 0.94. Abiyev [19] used a deep convolution neural network (CNN) to predict the possibility of lung lesions based on images, and classified 12 common diseases. However, most of the aforementioned models use a dataset based on a single standard image, and the strict requirements for objective data lead to a lack of flexibility in the method, wherein the patient’s complete medical record information cannot be used. Some key characteristic information in the medical record content, such as past history, current medical history, laboratory test results, and diagnosis, is lost, which makes these methods unsuitable to be directly applied to real medical records.

The development of natural language processing (NLP) and information extraction (IE) has immense application potential in the field of medical information [20, 21]. Electronic health records (EHRs) contain important information such as medical process data over the patient's entire course of the disease, including admission examination, methods used for examination, surgery, medication, and treatment effectiveness. Automatic semantic analysis of information can obtain effective information from unstructured text in EHRs, such as symptom set [22], contact history, adverse drug reactions [23], and diagnosis recommendation [24]. Sweidan [25], based on the fuzzy extended ER modeling model, used the unstructured content of EHRs to study liver fibrosis through entity extraction. Therefore, a neural network method based on EHR full text data can be used to predict the early stage of tuberculosis.

The initial clinical manifestations of tuberculosis are similar to those of pneumonia and lung cancer. In order to improve the accuracy of the model, a very large sample size is required. We must overcome the issue of a single image data source and adopt prediction based on comprehensive medical record information. Therefore, this study proposes a tuberculosis prediction model that uses structured as well as unstructured data to determine the possibility of tuberculosis infections. A multi-class data integration diagnosis model is used for prediction, so as to improve the efficiency of the prediction model.

## Methods

### Study design

In this study, the subjects were patients at the respiratory department and infectious cases department of a large comprehensive hospital in China between 2015 to 2020. The medical records used for inpatients include patient information, medical history, admission records, and medical laboratory examination and examination report. The medical records used for outpatients include outpatient information, outpatient treatment records, and medical laboratory examination and examination report.

**Fig. 1:**
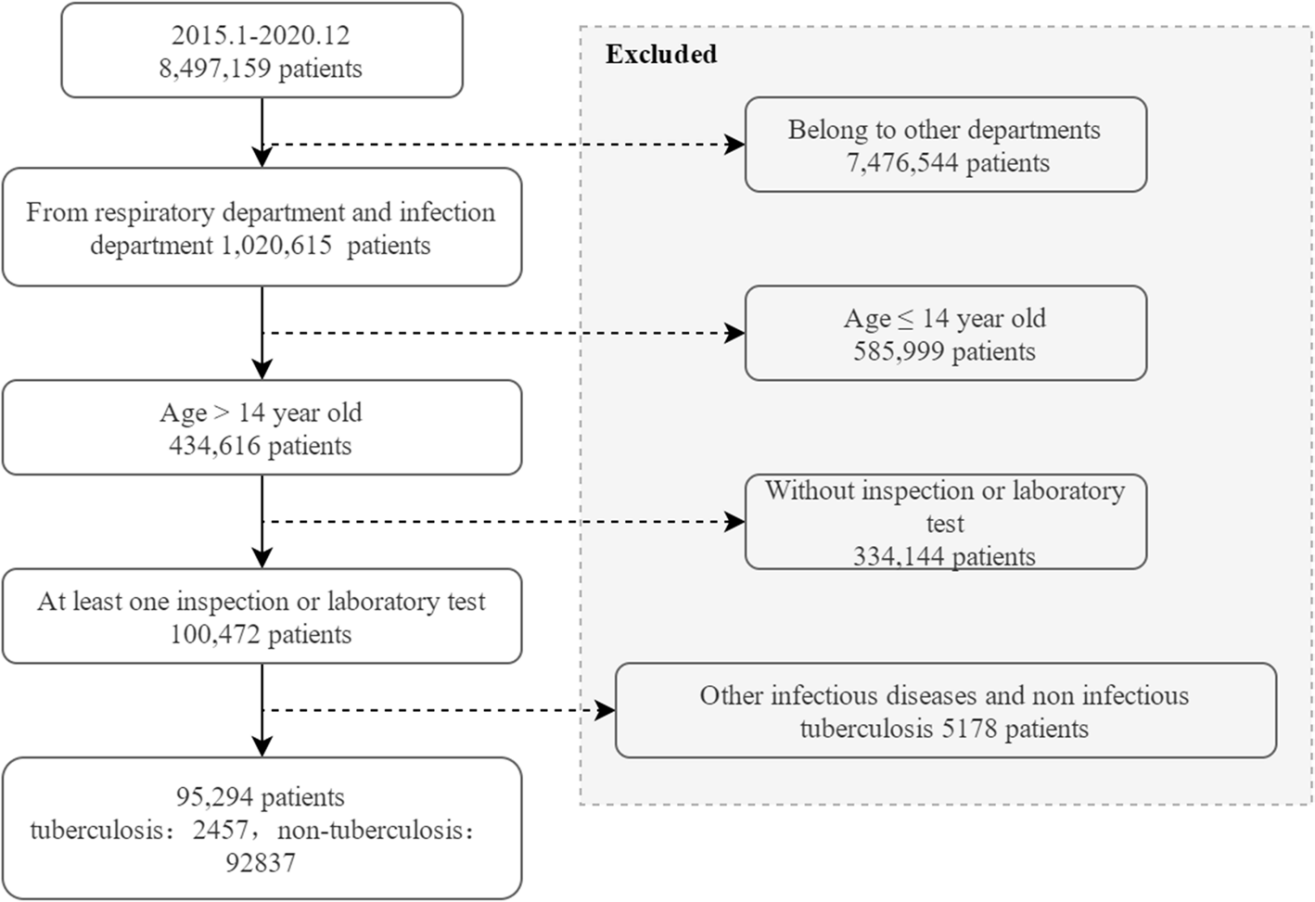
Flowchart of enrollment

First, we applied the quality control process to review the qualification of EHRs. Medical records with incomplete entries, inconsistent information, or follow-up medical records were discarded; 8,497,159 medical records remained. The dataset was then filtered according to the following inclusion criteria, as shown in Fig. 1: (1) The admission department is the respiratory department and infection-related department. (2) At least one inspection has been conducted in the hospital. (3) Those younger than 14 years are excluded. (4) Other categories of statutory infectious diseases are excluded. (5) Data related to tuberculosis but not infectious tuberculosis (such as obsolete pulmonary tuberculosis) is excluded. After screening, there were 95,294 medical records that met the criteria, with the average age being 46.92 years old. 48.89% were men and 51.11% were women.

The complete data were randomly divided into a training set and a verification set, and a fivefold cross-verification strategy was carried out using the classical 2–8 principle. In each fold experiment, the training set accounted for 80% and the test set accounted for 20%.

There are 2457 cases of pulmonary tuberculosis and 92,837 cases of non-pulmonary tuberculosis in this dataset. There is an obvious sample imbalance, which may easily lead to sample overfitting and make the model more inclined to incorrectly predict non-pulmonary tuberculosis. In this study, two methods were used to address this concern: down/oversampling and category weight. Random oversampling was conducted thrice for pulmonary tuberculosis samples with a small number of samples, and the sample size increased from 2457 to 7371 cases. Furthermore, the large number of non-tuberculosis samples were downsampled to 1/3 of the level, that is, they were downsampled based on the proportion of characteristics. For example, the sample was first divided into multiple sets according to the residential city, then downsampling was conducted 1/3 times from multiple sets to obtain multiple subsets, and finally multiple subsets were merged to obtain the final dataset. At this time, the proportion of residential cities in the dataset remained at the original proportion, which can reduce the negative impact of downsampling to a certain extent. Furthermore, category weight was added to increase the weight of tuberculosis samples and reduce the weight of non-tuberculosis samples in training. If the impact of a wrong prediction of tuberculosis is higher, the model will pay more attention to the prediction of tuberculosis category samples. Based on the parameter adjustment process, the category weight of pulmonary tuberculosis in this study was 8.5 and that of non-pulmonary tuberculosis was 0.75.

### Data processing

An electronic medical record contains two kinds of data, i.e., structured data such as gender, age or laboratory test results, and unstructured data in the form of free text written by doctors. Structured data is a key feature representation, and each field has a clear representative meaning. By contrast, free text represents the overall judgment of doctors on the current health status of patients and contains more comprehensive information. Based on the aforementioned characteristics of data, this study uses structured as well as unstructured data. On the basis of using the characteristics of structured data, this study uses unstructured data as a supplement to improve the prediction performance of tuberculosis.

**Fig. 2:**
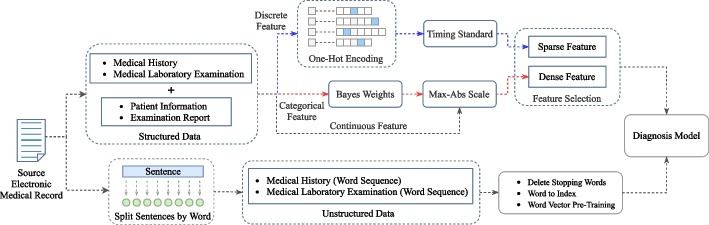
Technical infrastructure

In order to avoid the issue of label leakage when training the model, only documents such as Patient Information, Medical History, and Medical Laboratory Examination and Examination Report that can quantify the patient's physical indicators are used as model input data, and the data fields that clearly reflect the negative or positive etiology of pulmonary tuberculosis are filtered out. In addition, the diagnosis made by the doctor is used as the data label for model training. In the application process, the structured data are processed by feature engineering in many ways, including digitization, standardization, normalization, and feature screening. The technical process is illustrated in Fig. 2. For the use of unstructured text, specifically, the free text of patient cases is segmented according to the character level to obtain the word sequence, and then the words are counted to obtain a dictionary containing all words, in which each word is mapped into various serial number index values and finally applied to the model in the form of a word vector. At this time, the case information can be obtained from the free text without information loss, which helps overcome the concerns related to structured data. Finally, the two methods are combined, and the structured key features and full-text information are used to train the model concurrently, so as to make full use of the data to enhance the prediction performance of the model.

The study was approved by the Medical Science Research Ethics Committee of Peking University Third Hospital (serial number: IRB00006761-M2020318). All methods were performed in accordance with the relevant guidelines and regulations.

### Feature engineering

First, the data of each document and field are cleaned and encoded. One-hot encoding [26] is performed on discrete feature data. The outliers in continuous feature data are detected. For features having a clear value range, the outliers must be identified according to the value range and then replaced with the mean value of the feature. For example, the result of a laboratory examination cannot have a negative value. When a negative value appears, it will be regarded as an abnormal value. In addition, comprehensive use 3σ Based on the normal distribution theory, μ is the characteristic mean value, and σ is the characteristic standard deviation, with μ ± 3σ as the detection boundary. If the value is beyond the boundary, it will be regarded as an outlier and replaced with the mean value. After the above processing, the feature dimension of the data used in this research reached 68,313 dimensions. At this time, the dimension of data features is high, and high sparsity is caused by the existence of a large number of one-hot encoding features. When the input feature dimension in machine learning is high-dimensional sparse, the model is prone to over-fitting and poor generalization ability, resulting in better results in the training set and lack of accuracy in the test set. In order to solve the high-dimensional sparsity problem, a variety of feature engineering methods are used. This study uses two different strategies of algorithms: one is a single decision tree model, and the other is an integrated model. The integrated model selects random forests with relatively high variance and gradient boosting decision tree (GBDT) with relatively low deviations.

#### Time series standardization for history of present illness (HPI)

**Fig. 3:**
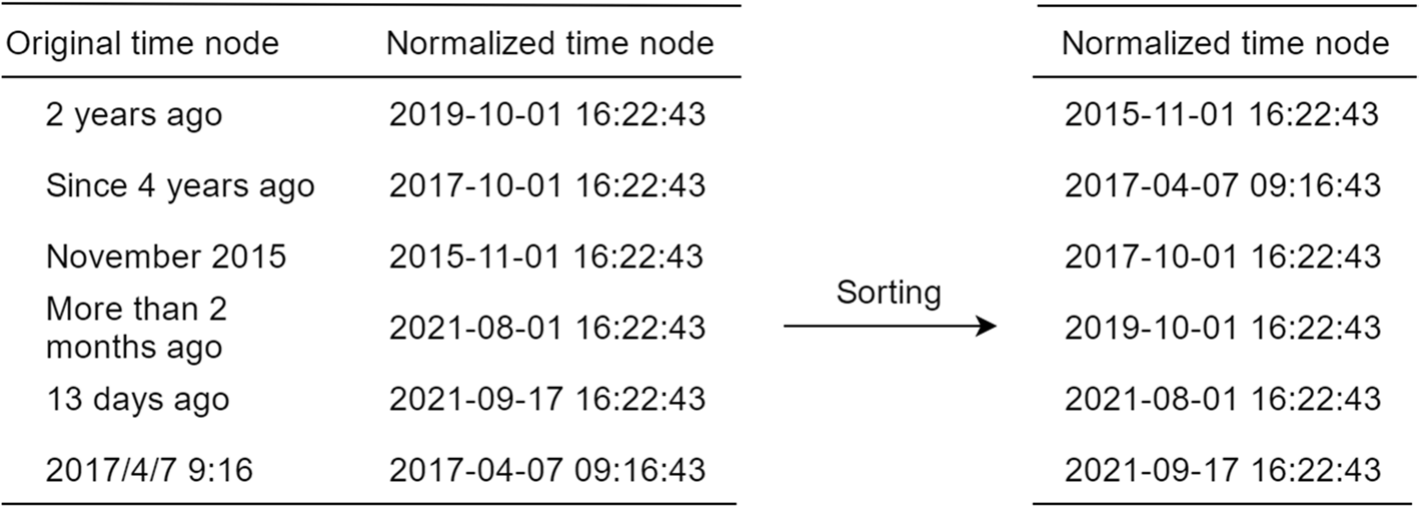
Time series standardization for HPI

In structured data, the time nodes stored in HPI have obvious time series characteristics. If the current medical history characteristics are directly one-dimensional, the original time-allowed characteristics will be lost. Time series standardization can effectively preserve the data characteristics of current medical history. Specifically, first the visit date of the current case is selected as the base date, and the negative and positive data of tuberculosis are clearly identified in the medical record or examination. Then the field is formatted about the time node in HPI and converted into the standard date format, and the time nodes are sorted from earlier to later, as shown in Fig. 3. Finally, the value of the feature in each time node is divided by the total number of time nodes of the case and multiplied by the ordinal number of time nodes where the feature is located for standardization. After this, order normalization is applied, with the weight of the feature closest to the current time being higher. After standardization based on time series, the model pays different amounts of attention to the features with different distance, and will either avoid paying too much attention to the features with long time distance or completely ignore them. As for the characteristics of near time, while retaining their relative importance, they will not be fully trusted. Furthermore, the sensitivity of this method to the features with short time distance is relatively stronger than that with long distance, and it also conforms to common sense logic.

The calculation process is given in Formula 1. Here, X_i_ represents the characteristic value of the i_th_ feature, T represents the total number of time nodes in the HPI of the sample, and t represents that the feature X_i_ appears at the t_th_ time node.

1 $${\mathrm{W}}_{\mathrm{i},\mathrm{t}}=\frac{\mathrm{t}}{\mathrm{T}}{\mathrm{X}}_{\mathrm{i}},\mathrm{ t}\in \mathrm{T}$$

#### Processing one-hot encoding feature based on Naive Bayesian method

Based on the outpatient and inpatient historical medical records, the characteristic information such as relevant symptoms, diseases, and signs under all main diagnoses are gathered. First, the logarithmic Bayesian probability value of each feature under the condition that the sample label is tuberculosis is calculated, and then the logarithmic probability of this feature under the condition that the sample label is not tuberculosis is calculated. Finally, the difference between the two is taken as the weight value of the feature relative to the disease. In tuberculosis prediction, the feature of each medical record is taken as the feature value of the corresponding feature based on the weight value calculated in the above steps. The feature value can reflect the correlation between the corresponding feature and the label, and improve the characterization ability of the feature while overcoming the issue of high-dimensional sparsity of the feature. The specific process is given in formula 2, where X_i_ represents the i_th_ feature, Y represents the sample label, Pulmonary Tuberculosis (PT) represents tuberculosis, and Healthy (HY) represents the health data of non-tuberculosis. W_i_ represents the i_th_ feature:

2 $$\begin{aligned}\mathrm P({\mathrm X}_{\mathrm i}\;\vert\;\mathrm Y\;=\;\mathrm{PT})&=\frac{\mathrm P(\mathrm Y\;=\;\mathrm{PT}\;\vert{\mathrm X}_{\mathrm i})\;\mathrm P({\mathrm X}_{\mathrm i})}{\mathrm P(\mathrm Y\;=\;\mathrm{PT})}\\ \mathrm P({\mathrm X}_{\mathrm i}\vert\mathrm Y=\mathrm{HY})&=\frac{\mathrm P(\mathrm Y\;=\;\mathrm{HY}\;\vert\;{\mathrm X}_{\mathrm i})\;\mathrm P({\mathrm X}_{\mathrm i})}{\mathrm P(\mathrm Y\;=\;\mathrm{HY})}\\W_i\;&=\;log(\mathrm P({\mathrm X}_{\mathrm i}\;\vert\;\mathrm Y\;=\;\mathrm{PT}))\;-\;\log(\mathrm P({\mathrm X}_{\mathrm i}\;\vert\;\mathrm Y\;=\;\mathrm{HY}))\end{aligned}$$

#### Finding feature subsets based on feature importance

Feature selection can eliminate irrelevant or redundant features, so as to reduce feature dimension, reduce feature data sparsity, improve model accuracy, and reduce running time [27]. Therefore, when we go to the feature input model, we often need to perform feature selection first to select and retain the features that are meaningful to the target task. At present, the most commonly used feature selection method is to use some machine learning models (such as tree model or integration method) to train, obtain the importance weight coefficient of each feature, and screen certain features according to the coefficient and set the threshold. However, as the internal structure and objective function of each model are different, there are differences in the selection of features. There is a certain deviation in the features selected by a single decision tree model. This study adopts the method of multiple models to select features together.

**Fig. 4:**
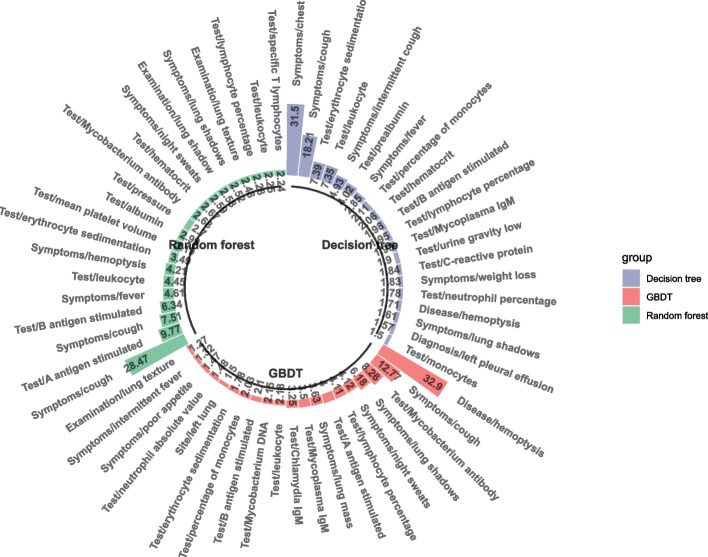
Top 20 feature names and corresponding importance weights of the three models

**Fig. 5:**
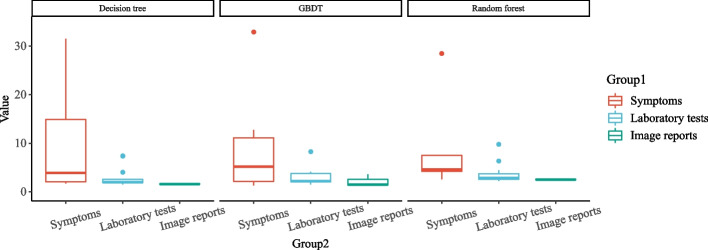
Proportion of symptoms in the characteristics is significantly higher than that in laboratory tests and imaging reports

The single decision tree model, random forest, and GBDT of the integrated model used in this study have diverse characteristics and can calculate feature importance more comprehensively. The importance weights of each feature in the three model results are sorted from high to low, as shown in Fig. 4. Then, the feature set is established, and the features are included in the feature exclusion set from high to low as per their weights. When the total importance of the set is higher than 0.7, the inclusion is stopped. Then, the three feature sets obtained by the three models are combined to obtain the final feature set. At this time, the features in the original feature set are filtered according to the result set to obtain the last used features (the sum of feature importance is higher than or equal to 0.7) for model training, as shown in Fig. 5. Based on the results of the importance of the characteristics of the three models, it can be observed that the proportion of main complaints is substantially higher than that of laboratory tests and image reports. Thus, a large amount of effective information exists in unstructured medical records.

After the feature processing, the following features will be obtained (partially listed in Table 1).

**Table 1:** Features after processing

Gender	Age	Symptoms / fever	Symptoms/ headache	Symptoms/ cough	Symptoms/ chest tightness	Symptoms/ chest pain
1	45	0.25	0.5	1	0	0
0	33	0.2	0.4	0	1	0
0	65	0.1	0.8	0	1	1

### Tuberculosis diagnosis model

**Fig. 6:**
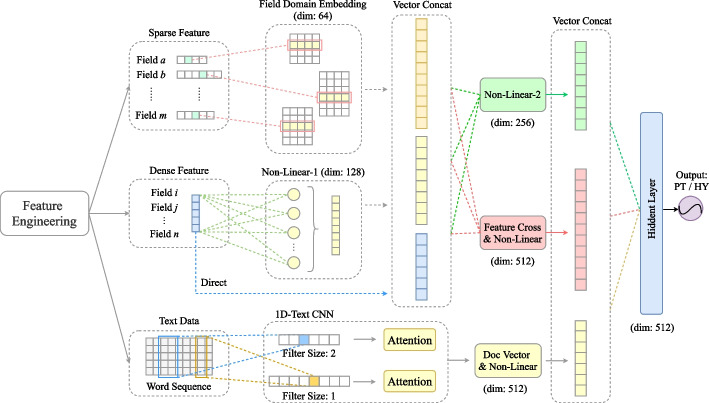
Multi-stream integration tuberculosis diagnosis model

In order to use the high-dimensional sparse feature to predict whether the patient has tuberculosis, in this study, a multi-stream integration tuberculosis diagnosis model (MSI-PTDM) is constructed, which can process sparse data, dense data, and unstructured text data concurrently. The model structure is shown in Fig. 6.

**Fig. 7:**
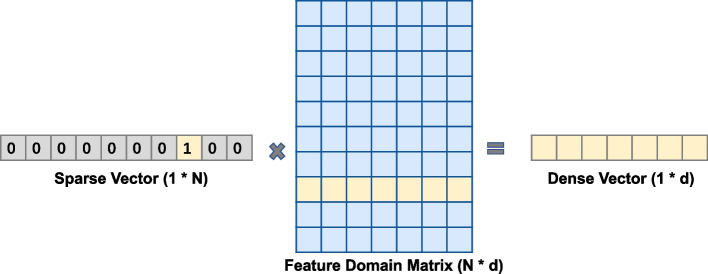
Process of converting a sparse vector into a dense vector

The structured field data in EHRs, such as structured test reports and basic information, is divided into two parts: sparse field data and dense field data. For example, with regards to the field 'check organ', there are many organs in the human body, which are discrete variables with a large number of categories, and the vector is sparse after one-hot coding. In processing sparse data, this model initializes a normally distributed dense eigen domain matrix for each field. In the calculation process, one-hot one-dimensional sparse vector and eigen domain matrix are multiplied to obtain a one-dimensional dense vector. This process transforms the discrete value of the original feature into an implicit vector represented by multiple dimensions. The specific process of converting a sparse vector into a dense vector is shown in Fig. 7, where N represents the dimension of the original sparse vector, d represents the dimension of the dense vector, and d is less than N. After the category features are converted through the one-hot method, each dimension of the sparse vector represents distinct feature values. The initialized feature domain matrix corresponds to a dense vector for each dimension of the sparse vector, so that the dense vector with dimension d is obtained after matrix multiplication. While reducing the feature dimension, the original one-hot sparse vector is changed into a dense vector. The feature domain matrix is trained in the process of subsequent binary classification model training. In addition, the dense field data is max absolute scaled and then spliced with multiple calculated one-dimensional dense vectors to form a field vector. Finally, linear operation and cross operation between features are performed on the field vector. The features after cross operation are incorporated into the model calculation process as new features to improve the nonlinear calculation ability of the model. The model not only calculates the influence of each feature on the prediction target, but also considers the combined interaction between features, so as to improve the feature learning space of the model and adapt to more complex feature interactive learning.

In addition, considering the possible accuracy and information loss in the process of structuring text data to obtain field data, this model also uses unstructured text data to extract the original data information and try to extract the hidden information contained in the text, which is used to make up for the information loss in the process of text data structuring to a certain extent. Specifically, the text data is segmented at the character level, and the document density vector carrying text information is calculated by using word embedding technology and a Text-CNN model constructed via one-dimensional convolution. Each dimension of the vector expresses the comprehensive information of the case text to some extent. At the end of the model, the dense vector result of the field feature flow is connected with the text dense vector, which is input into the shallow feedforward neural network. Then the sigmoid activation function is used to output whether it is the binary prediction result of pulmonary tuberculosis.

Field-based structured features are formal key information abstracted and extracted from free text. Each one-dimensional feature only represents one item of information in patient data and is the key point data. The task of medical diagnosis cannot be completely performed using a single feature, so the feature perception domain of the model should be expanded. Based on this idea, this research integrates feature interaction and unstructured text data. Feature interaction is the cross calculation between two features, after which further linear and nonlinear transformation is carried out through the network structure, which expands the scope of feature consideration. The unstructured text data is completed by Text-CNN, and finally the vector representing the entire case text is calculated. Given the existence of a convolution kernel, the receptive field of the model network layer continues to expand, and the text vector finally output has strong globality. This model uses field features or text word vectors concurrently, which not only makes up for the loss of information, but also takes into account the locality and globality of data, so as to support the model to better learn data and better complete the target diagnosis task.

The final results of the adjustable superparameters of the MSI-PTDM model proposed in this study are marked in the model diagram, and the specific parameter adjustment range is presented in the parameter optimization table, i.e., Table 2.

**Table 2:** MSI-PTDM parameter optimization

Field Domain Embedding	Non-Linear-1	Filter Size Combination	Non-linear-2	Feature-Cross	Doc Vector	Hidden-Layer
32	128	(1, 2)	32	128	256	64
64	256	(1, 3)	64	256	512	128
128	512	(1, 5)	128	512	1024	256
256	1024	(2, 3)	256	1024	2048	512
		(2, 5)	512	2048		1024
		(3, 5)	1024			

### Model evaluation

The model evaluation test set is used to evaluate the performance of four different models. In this study, four model evaluation indices were used, namely, sensitivity, specificity, accuracy, and AUC.

3 $$\begin{aligned}\mathrm{Sensitivity}\;\mathrm{SE}\;&=\;\frac{TP}{(TP+FN)}\\\mathrm{Specificity}\;\mathrm{SP}\;&=\;\frac{TN}{(TN+FP)}\\\mathrm{Accuracy}\;&=\;\frac{\left(\mathrm{TP}+\mathrm{TN}\right)}{P+N}\end{aligned}$$

True positive (TP) denotes the positive samples whose prediction answer is correct, true negative (TN) denotes the negative samples whose prediction is correct, false positive (FP) denotes the samples that are incorrectly predicted as positive, and false negative (FN) denotes the samples that are incorrectly predicted as negative.

## Results

In order to verify the superiority of the MSI-PTDM proposed in this study, we selected eight models for comparative experiments. In order to carry out an ablation experiment, we constructed a structured stream pulmonary tuberculosis diagnosis model (SS-PTDM) model that only retains the calculation flow of structured text data in MSI-PTDM and deleted the unstructured stream pulmonary tuberculosis diagnosis model (US-PTDM) of structured text data calculation stream in MSI-PTDM. The introduction of the above two ablation models makes it convenient to verify the prediction performance of the combined model of structured field data stream and unstructured text data stream. In addition, we select the commonly used models for text processing, such as Text Convolution Neural Network (Text-CNN) [28], Gated Recurrent Unit (GRU) [29], Long Short-Term Memory (LSTM), and Bi-directional Long Short-Term Memory (Bi-LSTM) [30] as a comparison to explore the performance of US-PTDM model under the condition of using only unstructured data. The Text-CNN model can focus more on local text information. By changing the filter size, it can calculate local information in various ranges. LSTM and GRU are good at describing text sequences. Bi-LSTM can calculate the positive and reverse order of text simultaneously. The aforementioned models have no restrictions on the length of input text sequences. They are classic models in the field of text prediction. In addition, we also select the machine learning model SVM, as well as the integrated models XGBoost [31] and random forest [32], to compare the prediction performance. SVM can perform different nonlinear calculations according to different kernel functions and is a common and effective model for machine learning; XGBoost is a model that has emerged in recent years and has achieved good results in many data competitions and industrial applications. The XGBoost, random forest, and SVM are used to predict the activity of Mycobacterium tuberculosis (Mtb) in tuberculosis and compare the prediction performance with our proposed SS-PTDM in structured data scenarios.

In order to conduct the comparative experiment fairly, we also optimized the parameters of the comparative model. For the Text-CNN model, a value within [1–5, 7, 9] is selected as the size alternative of the filter. On this basis, the number of combined stacked blocks of the convolution layer and the pooling layer is selected from [2, 4, 8, 12, 16], and average pooling and maximum pooling are the different choices of the pooling layer. The final optimal Text-CNN consists of four stacked blocks. The configuration of each block is Filter (5) + MaxPool, Filter (5) + MaxPool, Filter (3) + MeanPool, and Filter (3) + MaxPool.

For GRU, LSTM, and Bi-LSTM, Hidden_Size is selected from [128, 256, 384, 512, 1024]. For num_, the optional range of layers is [1, 2, 4, 6, 8, 16, 32], the value range of dropout is [0–0.5], and the step size is 0.05. The final optimal GRU has 16 layers, its Hidden_ Size is 512, and dropout is 0.2; the optimal LSTM has 8 layers, its Hidden_ Size is 384, and dropout is 0.2; the optimal parameter configuration of Bi-LSTM is 8 layers, its Hidden_ Size is 512, and dropout is 0.25.

Under the condition of structured data, the model is mainly optimized through GridSearch. For SVM, the kernel is selected from [rbf, poly], the value range of the regularization parameter C is [1–5], the step size is 0.5, and the gamma is selected from [0.01, 0.1, 1, 5]. When the kernel is poly, the degree is also searched from among [2–5]. Finally, the optimal parameter configuration of SVM is that the kernel is set to rbf, C = 1.5, and gamma is 1. For XGBoost, for max_, the deep selection range is [3–5, 7, 9], for learning_, the rate ranges from [0.05–0.3], and the step length is 0.05. In addition, it also includes a variety of super parameters. The final optimal XGBoost parameters are max_deep = 5, learning_rate = 0.15, n_estimators = 250, min_child_weight = 1, and gamma = 0.

Before model training, feature screening is required. First, the optimal threshold including the sum of feature importance is studied. Table 3 summarizes the experimental results of prediction using various thresholds and MSI-PTDM, where a threshold of 1 indicates no feature selection. When the sum of included feature importance is 0.7, the predicted result is in the optimal position. When the threshold is more than 0.7, each evaluation index decreases slightly. Consider the over-fitting of some samples caused by some too-sparse features. When the threshold is lower than 0.7, the reduction of various indicators is large, and some representative features are excluded.

**Table 3:** Experimental results of the sum of importance of different including features

Threshold	Accuracy	AUC	Sensitivity	Specificity
0	0.9591	0.9633	0.9227	0.9591
0.9	0.9588	0.9657	0.9227	0.9588
0.8	0.9602	0.9762	0.9273	0.9602
0.7	0.9691	0.9858	0.9318	0.9692
0.6	0.9325	0.9426	0.9101	0.9325
0.5	0.9000	0.9254	0.8636	0.9001

Based on the above threshold experiment, the total threshold of feature importance is selected as 0.7. Furthermore, a prediction experiment is carried out for the model under the fivefold cross validation. The average test results are summarized in Tables 4, 5 and 6. For each Fold in 1–5 Fold, the calculation formula used is:

4 $$1.96\times \sqrt{\frac{\mathrm{Accuracy}\times \left(1-\mathrm{Accuracy}\right)}{n},}$$

where n is the number of samples in the test set (n = 7663).

**Table 4:** Comparison of accuracy of 5-fold cross validation based on structured data models

	1-Fold	2-Fold	3-Fold	4-Fold	5-Fold	Mean	STD
SS-PTDM	0.9505 (0.9456, 0.9554)	0.9471 (0.9421, 0.9521)	0.9569 (0.9524, 0.9614)	0.9498 (0.9449, 0.9547)	0.9448 (0.9397, 0.9499)	0.9498 (0.9458, 0.9538)	0.0046
XGBoost	0.9337 (0.9281, 0.9393)	0.9301 (0.9244, 0.9358)	0.9305 (0.9248, 0.9362)	0.9285 (0.9227, 0.9343)	0.9298 (0.9241, 0.9355)	0.9305 (0.9288, 0.9322)	0.0019
Random Forest	0.8952 (0.8883, 0.9021)	0.8971 (0.8903, 0.9039)	0.8895 (0.8825, 0.8965)	0.9086 (0.9021, 0.9151)	0.9074 (0.9009, 0.9139)	0.8996 (0.8924, 0.9068)	0.0082
SVM	0.9376 (0.9322, 0.943)	0.9325 (0.9269, 0.9381)	0.9331 (0.9275, 0.9387)	0.9282 (0.9224, 0.934)	0.9242 (0.9183, 0.9301)	0.9311 (0.9266, 0.9356)	0.0051

**Table 5:** Comparison of accuracy of 5-fold cross validation based on unstructured data models

	1-Fold	2-Fold	3-Fold	4-Fold	5-Fold	Mean	STD
US-PTDM	0.9451 (0.94, 0.9502)	0.9485 (0.9436, 0.9534)	0.9434 (0.9382, 0.9486)	0.9431 (0.9379, 0.9483)	0.9452 (0.9401, 0.9503)	0.9451 (0.9433, 0.9469)	0.0021
Text-CNN	0.9284 (0.9226, 0.9342)	0.9205 (0.9144, 0.9266)	0.9111 (0.9047, 0.9175)	0.9151 (0.9089, 0.9213)	0.9175 (0.9113, 0.9237)	0.9185 (0.9128, 0.9242)	0.0065
GRU	0.9112 (0.9048, 0.9176)	0.9124 (0.9061, 0.9187)	0.9125 (0.9062, 0.9188)	0.9084 (0.9019, 0.9149)	0.9165 (0.9103, 0.9227)	0.9122 (0.9097, 0.9147)	0.0029
LSTM	0.9051 (0.8985, 0.9117)	0.9104 (0.904, 0.9168)	0.8916 (0.8846, 0.8986)	0.9088 (0.9024, 0.9152)	0.9074 (0.9009, 0.9139)	0.9047 (0.898, 0.9114)	0.0076
Bi_LSTM	0.9152 (0.909, 0.9214)	0.9135 (0.9072, 0.9198)	0.9158 (0.9096, 0.922)	0.9272 (0.9214, 0.933)	0.9185 (0.9124, 0.9246)	0.9180 (0.9133, 0.9227)	0.0054

**Table 6:** Comparison of accuracy of 5-fold cross validation of MSI-PTDM Ablation Experiment

	1-Fold	2-Fold	3-Fold	4-Fold	5-Fold	Mean	STD
MSI-PTDM	0.9676 (0.9636, 0.9716)	0.971 (0.9672, 0.9748)	0.9735 (0.9699, 0.9771)	0.9628 (0.9586, 0.967)	0.9732 (0.9696, 0.9768)	0.9696 (0.9657, 0.9735)	0.0045
US-PTDM	0.9451 (0.94, 0.9502)	0.9485 (0.9436, 0.9534)	0.9434 (0.9382, 0.9486)	0.9431 (0.9379, 0.9483)	0.9452 (0.9401, 0.9503)	0.9451 (0.9433, 0.9469)	0.0021
SS-PTDM	0.9505 (0.9456, 0.9554)	0.9471 (0.9421, 0.9521)	0.9569 (0.9524, 0.9614)	0.9498 (0.9449, 0.9547)	0.9448 (0.9397, 0.9499)	0.9498 (0.9458, 0.9538)	0.0046

In addition, the corresponding confidence interval is calculated for the overall result of cross validation, which is listed in Mean column. The calculation formula is:

5 $$\left[\mu -1.96\times \frac{\sigma }{\sqrt{n}},\mu +1.96\times \frac{\sigma }{\sqrt{n}}\right],$$

where μ is the mean value, σ is the standard deviation, and n is the number of samples in one experiment.

**Fig. 8:**
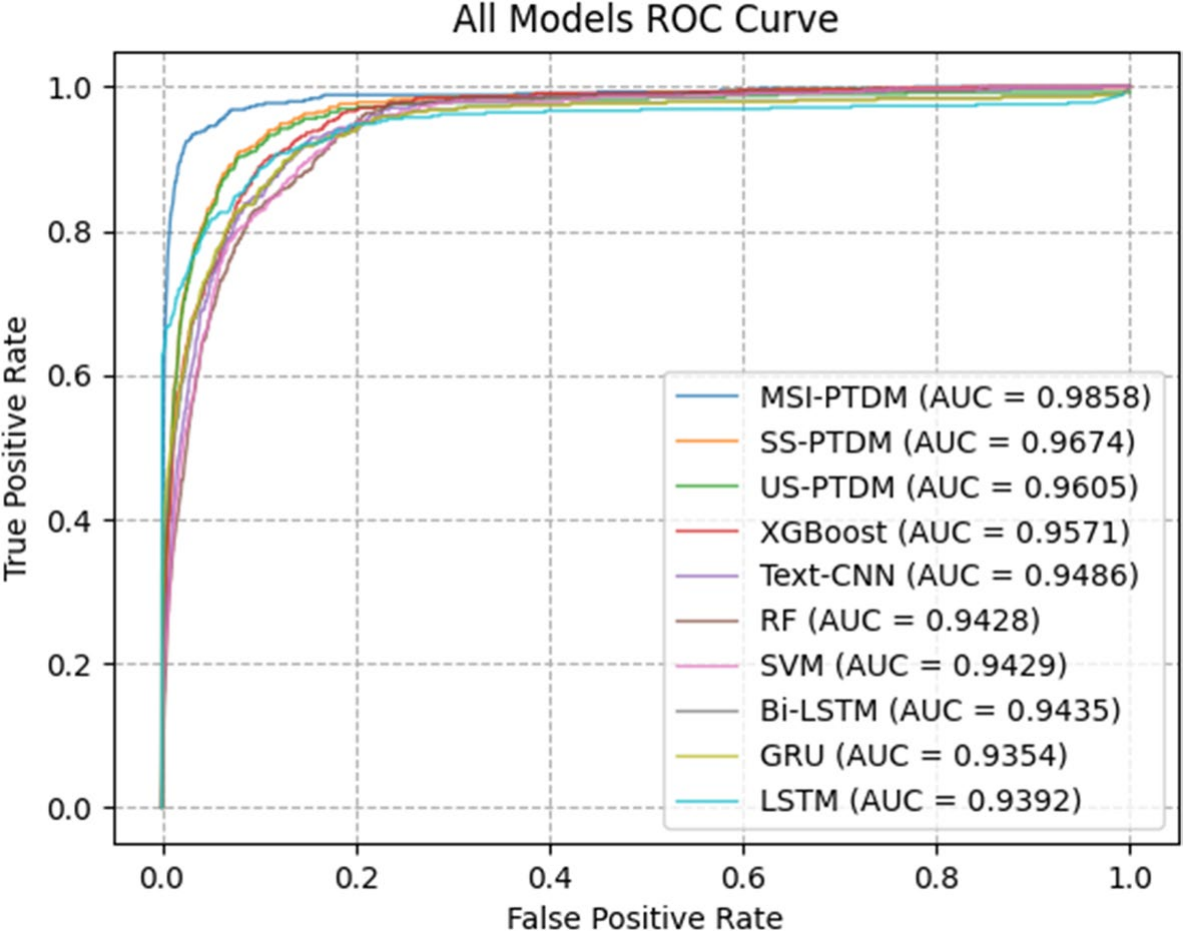
ROC of models

The final average test results are presented in Table 7, and the ROC curve is shown in Fig. 8. In the comparison of models using only structured data as input data and using unstructured data, SS-PTDM and US-PTDM models are better than other models, and the average accuracy is 0.9498 and 0.9451 respectively. In addition, in the ablation comparison experiment, the MSI-PTDM has the best performance in various evaluation indices, which is better than the single branch SS-PTDM and US-PTDM models, reflecting the advantages of the simultaneous application of structured data and unstructured data. The standard deviation of MSI-PTDM in the fivefold cross validation experiment is only 0.0044, which is generally lower than other models, indicating that the model also has good stability. In conclusion, this study proposes the optimal auxiliary task of MSI-PTDM for the diagnosis of pulmonary tuberculosis.

**Table 7:** Comparison of diagnostic accuracy between MSI-PTDM and other models of tuberculosis

	Accuracy	AUC	Sensitivity	Specificity
MSI-PTDM	0.9696 (0.9657, 0.9735)	0.9858 (0.9777, 0.9939)	0.9318 (0.9296, 0.9340)	0.9696 (0.9657, 0.9735)
SS-PTDM	0.9482 (0.9458, 0.9538)	0.9674 (0.9356, 0.9514)	0.8352 (0.8329, 0.8375)	0.9483 (0.9443, 0.9523)
US-PTDM	0.9453 (0.9433, 0.9469)	0.9605 (0.961, 0.9738)	0.8284 (0.8257, 0.8311)	0.9453 (0.9433, 0.9473)
Text-CNN	0.9185 (0.9128, 0.9242)	0.9486 (0.9513, 0.9697)	0.8251 (0.8225, 0.8277)	0.9186 (0.9130, 0.9242)
GRU	0.9122 (0.9097, 0.9147)	0.9354 (0.9414, 0.9558)	0.8208 (0.8164, 0.8252)	0.9122 (0.9095, 0.9149)
LSTM	0.9047 (0.898, 0.9114)	0.9292 (0.9244, 0.9464)	0.8142 (0.8081, 0.8203)	0.9047 (0.8981, 0.9113)
Bi_LSTM	0.9180 (0.9133, 0.9227)	0.9435 (0.9198, 0.9386)	0.8104 (0.8067, 0.8141)	0.9182 (0.9135, 0.9229)
XGBoost	0.9305 (0.9288, 0.9322)	0.9571 (0.9511, 0.9631)	0.8068 (0.8038, 0.8098)	0.9305 (0.9288, 0.9322)
Random Forest	0.8996 (0.8924, 0.9068)	0.9428 (0.9298, 0.9558)	0.8318 (0.8250, 0.8386)	0.8997 (0.8925, 0.9069)
SVM	0.9311 (0.9266, 0.9356)	0.9429 (0.9333, 0.9525)	0.7844 (0.7813, 0.7875)	0.9312 (0.9267, 0.9357)

## Discussion

### The disease characteristics of model screening are consistent with the guidelines

This study analyzed 20 characteristic indices related to tuberculosis, screened out disease-related factors, and established a diagnostic model for preliminary diagnosis and auxiliary diagnosis of tuberculosis. Some studies [33–37] showed that neutrophil to lymphocyte ratio (NLR), A\B antigen stimulated interferon detection, IGRA, and serum CA-125 were of definite significance in distinguishing tuberculosis from non-tuberculous pulmonary diseases. The features extracted from medical records in this study are consistent with the above studies. According to the standard of "WS 288–2017 diagnosis of tuberculosis" [38], the clinical symptoms, signs and related medical history, chest CT scanning findings, laboratory related examinations, and other clinical data of patients with initially diagnosed tuberculosis with negative etiology were classified into 12 observation indices. The characteristic results of tuberculosis obtained using decision tree, random forest, and GBDT are consistent with the criteria of WS 288–2017 pulmonary tuberculosis diagnosis such as *Mycobacterium tuberculosis* antibody test and TBAb; A \ B antigen stimulated interferon assay and IGRA; emaciation, night sweat, anorexia, cough, hemoptysis; and white globulin ratio and serum albumin globulin ratio (A/G). Lung shadows and the diagnostic criteria of pulmonary nodules were found in the findings. There are some features in the model that are not in the diagnostic criteria but are also meaningful for the diagnosis of tuberculosis, such as erythrocyte sedimentation rate and C-reactive protein. The increase of erythrocyte sedimentation rate is generally seen in various inflammatory diseases, tissue injury and necrosis, malignant tumors, and the relative or absolute increase of plasma globulin caused by various reasons [39]. The increase of fibrinogen and immunoglobulin significantly accelerated the erythrocyte sedimentation rate. The concentration of C-reactive protein in plasma increases rapidly and significantly in cases of acute myocardial infarction, trauma, infection, inflammation, surgery, and malignant tumors. It is a very sensitive index of acute phase response [40]. The concentration in plasma can reflect obvious inflammatory signals or the initiation stage of acute phase reaction, or chronic low-level inflammation and the beginning of acute phase reaction. Tuberculosis being an infectious disease, C reactive protein may also increase, but for different types of tuberculosis in different stages of disease, the C reactive protein manifestation needs further study. NLR is a common index to evaluate infection. The clinical value of NLR combined with lymphocytic reduction in the confirmation of bacteremia in an emergency is better than CRP, white blood cell count, and neutrophil count. NLR is of immense value in distinguishing bacterial pneumonia from tuberculosis, and is of considerable significance for early detection and early treatment of tuberculosis in primary hospitals [41, 42]. IGRA is mainly used to detect latent infections of tuberculosis, but is also used for the diagnosis, prediction, and differential diagnosis of active pulmonary tuberculosis [43]. IGRA is more accurate than TST in judging whether there is latent tuberculosis infection in patients with rheumatoid arthritis, especially for those who have been vaccinated with BCG vaccine and those infected with NTM [44]. This is of immense significance for the diagnosis and prevention of tuberculosis, especially in countries with high BCG vaccination rates.

### The model is applied to the diagnosis and treatment in hospitals

**Fig. 9:**
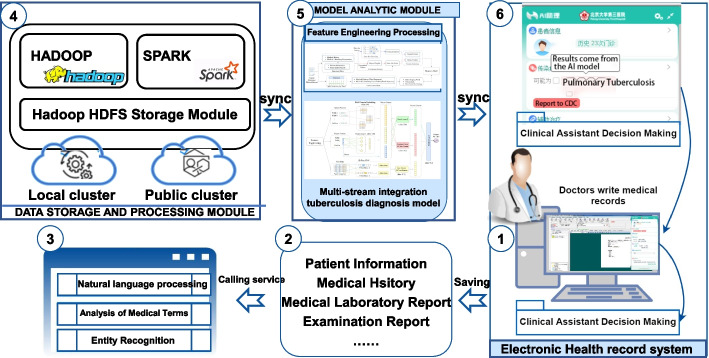
The architecture of combination of MSI-PTDM and EHRs

The proposed model was applied to the doctor workstation of a large general hospital in China to make predictions in real time while medical records were being written. The overall architecture is shown in Fig. 9. MSI-PTDM was installed upon a doctor workstation and operated in the real clinic for four months. When the doctor writes and saves the medical records at the doctor station, it will call the services. After the services and models deployed in the cloud complete their calculations, the results will be fed back to the doctor station. The experimental model was deployed and operated in the real environment of the hospital for 4 months, with 73 doctors from the respiratory department, emergency medical department, and thoracic surgery participating in the experiment (including 28 with senior professional titles and 45 with intermediate professional titles). Considering the respiratory department as an example, every day, 12 doctors in the respiratory department receive 30 patients in the outpatient and emergency department. A total of 692,949 patients were treated by 73 doctors in 4 months. The detailed records of the department's diagnosis and diagnosis of pulmonary tuberculosis are presented in Table 8. Hospital doctors’ computers are uniformly configured, the configuration being as follows: DELL desktop: Intel (R) core (TM) i7-7500u CPU @ 2.70Ghz, 2.90 GHz, running memory 8G, hard disk 256G. The average time to predict the presence of pulmonary tuberculosis from a complete medical record is 10 s.692949 patients were monitored, including 484 patients with confirmed pulmonary tuberculosis. MSI-PTDM predicted 440 cases of pulmonary tuberculosis. The positive sample recognition rate was 90.91%, the false positive rate was 9.09%, the negative sample recognition rate was 96.17%, and the false negative rate was 3.83%.

**Table 8:** Receiving department and confirmed pulmonary tuberculosis record

Department	2021–6	2021–7	2021–8	2021–9	Total	Confirmed patients with TB	Number of alerts
Respiratory department	9142	11137	11199	10629	42107	430	405
Emergency department	168362	145150	144951	166559	625022	39	27
Thoracic Surgery	6880	6191	7126	5623	25820	15	8
Total					692949	484	440

This study is the first to research tuberculosis prediction based on EHR text using artificial intelligence. It also integrates the model into doctor workstations and applies it in clinical work, which suggests the potential for text-based prediction of other diseases in the future.

## Limitations

There are some limitations in this study. 1. Only patients from the respiratory department and infectious cases department have been considered in this study, and the results are different from other departments. 2. Machine learning based on medical records needs medical records having higher quality, and the overall integrity of inpatient medical records must be better, including detailed records such as examination and inspection over a period of time. For medical records with fewer outpatient first visits or lesser medical record information, such as follow-up medical records, the prediction results may be inaccurate. 3. In view of the comprehensive limitations of the medical record template, some factors do not reflect significant differences, such as gene data. 4. For the time series standardization used for current medical history, the order of different time nodes is considered. However, the size of the time interval is not considered. 5. MSI-PTDM only uses structured field features and unstructured text data. In the future, more data streams must be integrated, such as patient image data, which will enable a more objective and three-dimensional prediction by the model.

## Conclusions

A neural network is designed based on multi-stream EHR data for the preliminary diagnosis of tuberculosis. First, specific and detailed feature engineering is performed for the structured data, and the feature selection methods of multiple models is adopted. It also puts forward features that are not covered in the standard of "WS 288–2017 diagnosis of tuberculosis" such as erythrocyte sedimentation rate and C-reactive protein, but are of considerable significance for diagnosis. Based on the high-dimensional sparse features, a multi-stream integrated diagnosis model that can concurrently process sparse field data, dense field data, and unstructured text data is constructed. Medical sparse features are embedded with feature domain vectors, and single-valued sparse vectors are represented by multi-dimensional dense hidden vectors. Simultaneously, the side effects of sparsity on model training are alleviated. MSI-PTDM is more suitable for the target research of current medical data as it involves the specific time series standardization of current medical history. However, structural features are extracted from the text, and it will suffer from information loss. Adding the processing of the original unstructured text makes up for the error of the aforementioned process to a certain extent, and takes into account the local and global data, so that the model can learn the data more comprehensively and effectively. In addition, MSI-PTDM also introduces the interaction between features, considers the combination effect between patient features, adds more complex nonlinear calculation considerations, and improves the learning ability of the model. It has been verified in the test set and real environment deployment. Using the indicators of sensitivity, specificity, accuracy, and AUC, MSI-PTDM was compared with SS-PTDM, XGBoost, Text-CNN, Random Forest, GRU, LSTM, Bi-LSTM, and SVM without an unstructured text processing data stream and achieved better results. MSI-PTDM was also installed in the respiratory clinic of a large general hospital in China. Combined with the doctor's medical record system, the accuracy of real-time prediction of tuberculosis was 90.91%. These results show that this method has high accuracy and can be applied to the auxiliary decision-making of pulmonary tuberculosis in medical scenarios to reduce the probability of missed diagnosis.

## Data Availability

The data that support the findings of this study are available from the corresponding author upon reasonable request.

## Abbreviations

EHRs: Electronic health records
MSI-PTDM: Multi-stream integration tuberculosis diagnosis model
GBDT: Gradient boosting decision tree
HPI: History of present illness
TP: True positive
TN: True negativeFPFalse positiveFNFalse negative
AUC: The area under the receiver operating characteristic curve
SS-PTDM: Single stream tuberculosis diagnosis model
Text-CNN: Text Convolution Neural Network
GRU: Gated Recurrent Unit
LSTM: Long Short-Term Memory
Bi-LSTM: Bi-directional Long Short-Term Memory
